# Correction: Advancing the measurement of knowledge, attitudes and practices of health workers who care for women and girls who have undergone female genital mutilation/ cutting (FGM/C): A qualitative exploration of expert opinion

**DOI:** 10.1371/journal.pone.0291509

**Published:** 2023-09-08

**Authors:** The PLOS ONE Staff

## Notice of republication

This article was republished on August 30, 2023, to correct the copyright statement to reflect the WHO-affiliated copyright. The publisher apologizes for the errors. Please download this article again to view the correct version. The originally published, uncorrected article and the republished, corrected articles are provided here for reference.

## Supporting information

S1 File Originally published, uncorrected article. (PDF)

S2 File Republished, corrected article. (PDF)
