# Supplementary material for: Correction: Advancing the measurement of knowledge, attitudes and practices of health workers who care for women and girls who have undergone female genital mutilation/ cutting (FGM/C): A qualitative exploration of expert opinion
Source: PLoS One. 2023 Sep 8;18(9):e0291509. doi: 10.1371/journal.pone.0291509 (PMC10490967; doi:10.1371/journal.pone.0291509)
Supplement: S2 File — (PDF) [file pone.0291509.s002.pdf]

PLOS ONE

RESEARCH ARTICLE

# Advancing the measurement of knowledge, attitudes and practices of health workers who care for women and girls who have undergone female genital mutilation/ cutting (FGM/C): A qualitative exploration of expert opinion

**Christina X. Marea**[1]*, **Nicole Warren**[2☯], **Nancy Glass**[2‡], **Wisal Ahmed**[3‡], **Christina C. Pallitto**[3☯]

**1** Georgetown University School of Nursing, Washington, D.C., United States of America, **2** Johns Hopkins University School of Nursing, Baltimore, MD, United States of America, **3** UNDP-UNFPA-UNICEF-WHO-World Bank Special Programme of Research, Development and Research Training in Human Reproduction (HRP), Department of Sexual and Reproductive Health and Research, World Health Organization, Geneva, Switzerland

☯ These authors contributed equally to this work.
‡ NG and WA also contributed equally to this work.
* christina.marea@georgetown.edu

Check for updates

OPEN ACCESS

**Citation:** Marea CX, Warren N, Glass N, Ahmed W, Pallitto CC (2023) Advancing the measurement of knowledge, attitudes and practices of health workers who care for women and girls who have undergone female genital mutilation/ cutting (FGM/C): A qualitative exploration of expert opinion. PLoS ONE 18(4): e0284900. https://doi.org/10.1371/journal.pone.0284900

**Editor:** Joyce Jebet Cheptum, National Defence University - Kenya, KENYA

**Received:** May 11, 2022

**Accepted:** April 1, 2023

**Published:** April 27, 2023

**Copyright:** © 2023 World Health Organization. Licensee Public Library of Science. This is an open access article distributed under the Creative Commons Attribution IGO License, which permits unrestricted use, distribution, and reproduction in any medium, provided the original work is properly cited. https://creativecommons.org/licenses/by/3.0/igo/. In any use of this article, there should be no suggestion that WHO endorses any specific organization, products or services. The use of the WHO logo is not permitted. This notice should be preserved along with the article's original URL.

**Data Availability Statement:** Data cannot be shared publicly because the sample for this qualitative study is a very small and specifically defined group. The transcripts include content that

## Abstract

### Background

Female genital mutilation or cutting (FGM/C) is a social norm driven practice associated with numerous adverse health complications. Existing assessment tools for health workers are limited by lack of a clear framework for what constitutes the critical knowledge, attitudes, and practices that impact FGM/C prevention and care. The aim of this study was to explore expert opinion of the knowledge, attitudes, and practices for FGM/C-related prevention and care that can be used to inform the development of future KAP measurement tools.

### Methods

We conducted 32 semi-structured individual interviews with global clinical and research experts on FGM/C from 30 countries including participants from Africa, Australia/ New Zealand, Europe, the Middle East, and North America. Interview questions explored areas of knowledge, attitudes, and practices that influence FGM/C-related prevention and care activities. We used the directed content analysis methodology for the qualitative data analysis.

### Results

We identified six categories of knowledge, six of practice, and seven of attitudes that contribute to FGM/C-related prevention and care. Areas of knowledge included: general knowledge about FGM/C; who is at risk for experiencing FGM/C; support for FGM/C; female

genital anatomy/ physiology; health complications of FGM/C; management of health complications of FGM/C; ethical and legal considerations for the treatment and prevention of FGM/C, and patient-health worker communication. Areas of practice included: clinical procedures and protocols; management of complications; defibulation; other surgical procedures for FGM/C; pediatric care (including prevention); and patient-centered care. Participants described health worker attitudes that may affect how prevention and care activities are delivered and/or received including attitudes toward: the perceived benefits of FGM/C; harms of FGM/C; ethical considerations related to FGM/C medicalization, prevention, and treatment; providing care for FGM/C-affected clients; women and girls who have experienced FGM/C; communities that practice FGM/C; and affective response to FGM/C. We also present participant perspectives on the ways in which knowledge, attitudes, and practice interact impacting the type and quality of care provided to those affected by FGM/C.

## Conclusions

This study identified specific areas of knowledge, attitudes, and practices in FGM/C prevention and care that are important to include in future evaluation metrics. Future KAP tools should be theoretically informed using the framework we present, and assessed for validity and reliability using psychometrically rigorous methods. Developers of KAP tools should consider the hypothesized relationships between knowledge, attitudes, and practices.

would make participants identifiable. Data are available from the Johns Hopkins Medicine Institutional Ethics Committee (contact via jhmeirb@jhmi.edu) for researchers who meet the criteria for access to confidential data.

**Funding:** This work received funding from the UNDP-UNFPA-UNICEF-WHO-World Bank Special Programme of Research, Development and Research Training in Human Reproduction (HRP), a co-sponsored programme executed by the World Health Organization (WHO). The named authors alone are responsible for the views expressed in this publication and do not necessarily represent the decisions or the policies of the UNDP-UNFPA-UNICEF-WHO-World Bank Special Programme of Research, Development and Research Training in Human Reproduction (HRP) or the World Health Organization (WHO). Research reported in this publication was also supported by the National Center For Advancing Translational Sciences of the National Institutes of Health under Award Number TL1TR001431 and KL2TR001432. The content is solely the responsibility of the authors and does not necessarily represent the official views of the National Institutes of Health. This work was also supported by the Rockefeller University Heilbrunn Family Center for Research Nursing through the generosity of the Heilbrunn Family. The funders had no role in study design, data collection and analysis, decision to publish, or preparation of the manuscript.

**Competing interests:** They authors have declared that no competing interests exist.

**Abbreviations:** FGM/C, female genital mutilation/ cutting; WHO, World Health Organization; KAP, knowledge, attitudes, and practices.

## Background

Female genital mutilation/ cutting (FGM/C) is a persistent public health problem which has already affected approximately 200 million women and girls worldwide, and another 3 million are at risk each year [1]. Despite the prevalence of FGM/C and the global commitments and national laws promoting its abandonment, health workers in a variety of settings may not be adequately prepared to provide quality evidence-based prevention and treatment of FGM/C complications [2–8]. FGM/C is a harmful practice performed on girls from infancy to adolescence, depending on the setting, and involves partial or total removal of external female genitalia [1]. FGM/C is concentrated in 30 countries in the Middle East, Africa and Asia; however, due to migration, women and girls at risk for FGM/C, or who have undergone this practice, live around the world [9–12]. Although prevalence rates are falling in many countries, population growth data suggest that the total number of affected women and girls will continue to rise for several decades unless urgent action is taken to prevent it [13].

This growing population requires specialized care because FGM/C is associated with acute and chronic health complications negatively affecting genitourinary, obstetric, gynecologic, sexual, and mental health, and results in a global yearly estimated cost of 1.4 billion US dollars [14]. Health workers must understand their role in preventing and treating FGM/C to address the tremendous health burden and economic costs. Knowledge, attitudes, and practices (KAP) assessments have been developed to better understand health worker preparedness for FGM/C prevention and care, to develop capacity building interventions, and for monitoring and evaluation purposes. However, a systematic review highlights that the development of these KAP tools have often not been guided by a systematic process, and that they have gaps in terms of their validity in assessing clinical care and decision making [15]. Findings from this review informed the current study, the aim of which is to identify and understand the knowledge,

attitudes, and practices considered most important for FGM/C-related prevention and care. These findings will result in a comprehensive bank of items that will inform the development of a validated KAP measurement tool being developed by the World Health Organization (WHO).

The *Knowledge*, *Attitudes*, *and Practices (KAP)* framework theorizes that an individual learns about a topic (knowledge), develops some affective response (attitude), and engages in a behavior (practice)–often these factors influence one another in multidirectional ways [16, 17]. The KAP framework addresses the relationship between knowledge and behavior, theorizing that attitudes may affect the relationship [16]. KAP tools are often used to assess health behaviors, including the behaviors of health workers during the provision of care. In the context of FGM/C, broad domains of knowledge, attitudes, and practices have emerged in existing evidence-based guidelines and a review of existing KAP tools [15, 18]. Knowledge may include awareness of the practice, its health implications, and associated care. Attitudes may include the affective responses of the health worker that affect what prevention or care services are offered, and the manner in which they are delivered. Practices may include health care worker behaviors as they operationalize their knowledge for the prevention and care of patients affected by FGM/C. Some existing studies suggest that knowledge changes attitudes and then attitudes will change behavior, in that order; however, the sequencing of these elements can be highly variable and not follow a linear trajectory. For example, behavior change may lead to attitudinal changes and then, finally, increased knowledge.

Multiple authors have used a KAP framework to guide their research assessing the KAP of health workers who care for those affected by FGM/C, including two recent systematic reviews, which found that health workers are ill prepared to provide FGM/C-related prevention and care [19, 20]. The status of health workers knowledge, attitudes and skills is reflected in their experience providing care. Studies have demonstrated that health workers report challenges providing care to people affected by FGM/C in both low and medium/ high prevalence settings including significant knowledge deficits and low confidence in their ability to provide quality care for clients affected by FGM/C [21–23].

Existing FGM/C KAP tools have limited comparability due to the inclusion of widely different items and response formats across studies, despite similar aims of assessing health worker KAP for FGM/C. This variability may reflect that existing FGM/C KAP tools were not informed by formative qualitative research, and few underwent psychometric assessment [15, 24]. During instrument development, researchers should conduct qualitative research to develop the concepts of interest and understand the priorities of a population prior to attempting to develop items for a quantitative measure [25, 26]. In the absence of this qualitative research, measures may not adequately reflect how users perceive a given phenomenon. Formative qualitative research guides item development and subsequent psychometric testing to assesses the reliability and validity of the resulting measure. An FGM/C KAP tool, informed by qualitative research, and eventually psychometric testing, would permit a meaningful evaluation of health worker KAP, changes over time, and comparisons across populations. Such a tool is increasingly important as the absolute number of women and girls affected by FGM/C grows.

## Methods

### Sample

We developed a purposive sampling strategy that included stratified enrollment of 6–8 experts from each of the identified clinical and/ or research specialties (reproductive health, primary care/ pediatrics, nursing, public health/ social science) for a preliminary aim to enroll a total of

24–32 participants, evenly split across high and low prevalence settings. We used the UNICEF definitions of high and low prevalence such that those countries where FGM/C has been practiced endemically by >10% of the population (approximately 30 countries) are classified as high prevalence [1]. The low prevalence countries according to the UNICEF definition predominantly include those where FGM/C is present among immigrants/ migrants from high prevalence countries.

**Recruitment and consent.** We began our recruitment process by generating a list of clinical and research experts across our enrollment strata, and then populated it with experts identified via research authorship, clinical expertise, FGM/C networks, and referrals from study team members. Our initial list included 39 clinical and research experts, and an additional 9 individuals were referred by those contacted for a total of 48 individuals who were invited to participate. Of the 48 individuals invited to participate, 31 accepted and completed an interview. We invited 21 from the high prevalence region of whom 14 (66%) completed an interview, and 27 from the low prevalence countries of whom 17 (63%) completed an interview. We planned for equal enrollment between the two regions; however, in order to ensure saturation of themes in the low prevalence countries we needed to add additional interviews as new themes emerged during interviews #13 and #14 for that region.

We identified FGM/C clinical and research experts across our predefined strata as demonstrated by clinical practice and/or publication and research history, possession of a clinical or research degree, and the ability to conduct an interview in English or French. We contacted potential participants via email. Those willing and eligible to enroll were scheduled for an interview via their preferred modality—in-person, via telephone, or through secure internet-based voice calls. We also requested referrals to identify sufficient numbers of participants in each strata. We continued with directed outreach to potential participants until we had completed adequate interviews across all strata and achieved data saturation.

**Setting and participants.** Interviews were conducted in English or French (via interpreter) between September 2018 and January 2019 by author CM, a female nurse-midwife and PhD candidate experienced in FGM/C clinical care with training in qualitative research. The interview began by CM introducing her clinical and research background, explaining the study's purpose and procedures, and obtaining verbal informed consent. Interviews were conducted at a time and place, and using a format acceptable and convenient to the participants. Interviews were audio-recorded with participants' permission and lasted from 45–150 minutes. Two participants provided written responses due to poor audio connection.

## Interview guide

We organized our interview guide according to our a priori domains of *Knowledge*, *Attitudes*, *and Practices*. The interview guide explored the participant's opinion of: 1) the knowledge required to provide quality care for women and girls affected by FGM/C; 2) the attitudes that health workers may hold related to FGM/C and the care of FGM/C-affected women and girls; and 3) the clinical practices necessary for the provision of high-quality health care for women and girls affected by FGM/C.

## Analysis

We used a directed content analysis approach to analyze the data via the coding and identification of themes using a systematic classification process [27]. Directed content analysis is a useful methodology when conducting research about a phenomenon that requires further examination and clarification. This approach can validate and/ or conceptually extend a theoretical framework–in this case the KAP framework in the context of FGM/C.

Following the interviews, audio recordings were immediately transferred from the recording device to a secure server hosted by Johns Hopkins University and erased from the recording device. Audio recordings were transcribed verbatim and error checked by a certified transcription and translation company. Written comments submitted in French (n = 2) were translated into English for analysis. We used NVivo 12 software to manage our analysis. Co-authors CM and NW led the qualitative analysis, and began by developing a preliminary codebook based on an a priori framework compromised of existing knowledge, attitudes, and practices categories published in a recent systematic review assessing the content of existing KAP measures [15]. Codes represented a topical area of interest or a specific example of that topic. CM and NW independently coded the same interview using the preliminary codebook and added additional codes as necessary for content that did not align with the a priori framework. CM and NW then compared coding and wrote a brief summary of each code. They then independently coded another three of the same interviews, then compared their coding engaging in discussion to reach at least 80% consensus in coding, refining the code summaries as needed. They then applied the finalized codebook to all interviews.

### Ethics statement

The Institutional Review Boards of Johns Hopkins Medicine (IRB00159147) and the World Health Organization (ERC.0003031) granted ethical approval for the conduct of this study. We obtained oral consent from all participants.

## Results

We conducted a total of 31 in-depth interviews with 14 participants from high/medium prevalence countries and 17 participants from low prevalence countries. Four interviews were conducted in-person, twenty-five by phone or secure voice-over-internet-protocol (VOIP), and two provided written responses due to limitations of phone/ internet connection. Table 1 describes the sample by region and profession.

Table 2 describes the participant characteristics. The participants were mostly female (77%). Nearly half of participants held a PhD (48%), while the remainder held a clinical or master's degree. The most common religious affiliations were Muslim (10%), None (30%) and Christian (27%). There was a wide range of ages (31–77 years old), and years conducting clinical or research work related to FGM/C (6–55 years).

We identified six categories of knowledge, six categories of attitudes, and seven categories of practices. Each category is comprised of several codes that describe topical areas and illustrative examples provided by participants. See Figs 1–3 for an overview of codes and categories for knowledge, attitudes, and practices, respectively. Codes that were specific to low or high/ medium prevalence settings are indicated in the figures. In the narrative that follows, we provide additional context to the figures, and explore the ways in which the KAP elements interact to inform prevention and care.

**Table 1. Qualitative interview participants–region and profession.**

| | Reproductive Health workers | Primary Care/Pediatric | Nursing | Researchers | TOTAL |
|---|---|---|---|---|---|
| High/ Medium Prevalence | 4 | 4 | 3 | 3 | 14 |
| Low Prevalence | 6 | 3 | 3 | 5 | 17 |
| TOTAL | 10 | 6 | 6 | 8 | 31 |

https://doi.org/10.1371/journal.pone.0284900.t001

**Table 2. Participant characteristics.**

(n = 31)

| Characteristic | N (%) | | |
|---|---|---|---|
| **Region of Origin** | | | |
| • High Prevalence | 14 (45%) | | |
| • Low Prevalence | 17 (55%) | | |
| **Highest Level of Education** | | | |
| • PhD* | 15 (48%) | | |
| • MD* | 12 (39%) | | |
| • Masters | 3 (10%) | | |
| • DNP** | 1 (3%) | | |
| **Religion** | | | |
| • Muslim | 10 (33%) | | |
| • Christian | 8 (27%) | | |
| • Jewish | 2 (7%) | | |
| • None | 9 (30%) | | |
| • Decline | 1 (3%) | | |
| | **Range** | **Mean** | **Standard Deviation** |
| **Age** | 31–77 | 51.8 | 11.3 |
| **Years in Profession** | 5–55 | 20.8 | 10.85 |
| **Years in FGM/C Related Work*** ** | 6–55 | 21.4 | 13.6 |

*One participant reported MD/ PhD

**DNP = Doctor of Nursing Practice

***3 missing values

https://doi.org/10.1371/journal.pone.0284900.t002

## Codes and categories in context

In this section, we provide additional context for the codes and categories represented in Figs 1–3 to clarify their relationship to health worker KAP. First, foundational knowledge of female anatomy and physiology is a prerequisite for providing care to people with FGM/C. This includes being able to recognize variations of external genitalia including clitoral size and function. For example, by recognizing which structures were affected by cutting, the health worker may take a more relevant history and identify possible treatments to control symptoms. This information was considered critical for all members of the health care team, not only physicians:

> *". . . some categories of midwives. . . [do] not know definitely the physiology and anatomy of these genital parts so they need to concentrate in raising their knowledge about this. . ." (High Prevalence)*

Requisite knowledge of anatomy and physiology factors that influence sexual function, such as arousal, desire, and relationship characteristics, were also deemed critical for providing care that would comprehensively address FGM/C's sequalae.

When it came to FGM/C-specific content, participants agreed that understanding FGM/C types per the WHO definition and key terms such as defibulation (the surgical opening of the closed genital scar resulting from type III FGM/C) and FGM/C medicalization (when FGM/C is practiced by any category of health worker any time in a woman or girl's life) were considered important [18]. All participants stressed the importance of understanding risk factors for

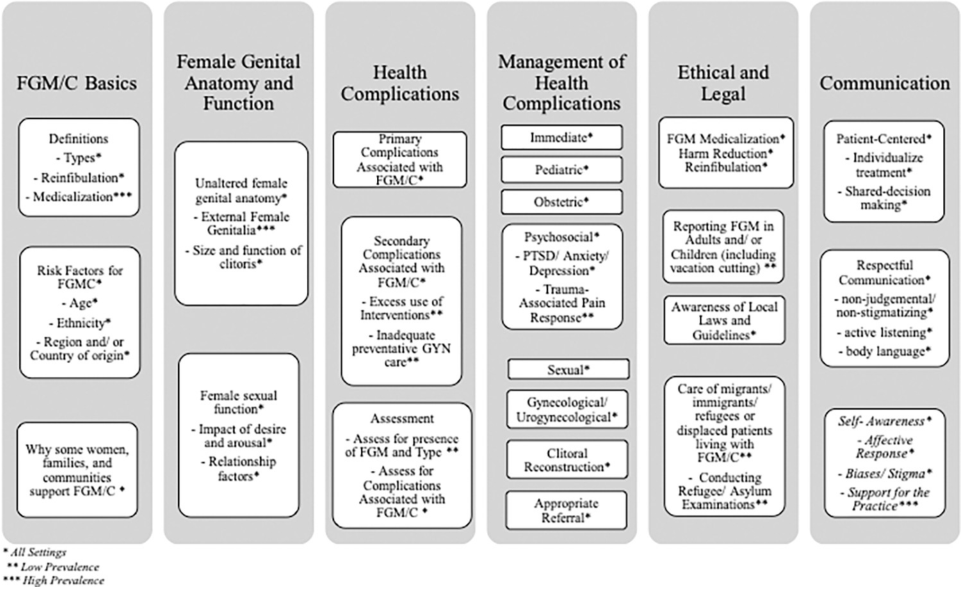


**Fig 1. Knowledge: Categories and codes.** *All Settings, ** Low Prevalence, ***High Prevalence.

https://doi.org/10.1371/journal.pone.0284900.g001

FGM/C such as age, ethnicity and country of origin- as well as motivation for the practice. Although these motivations can vary by setting, participants articulated a clear need for health workers to recognize them. Several health workers pointed to key resources to do this: the WHO *Guidelines for the Management of Health Complications From FGM* and WHO's *Care of girls and women living with female genital mutilation*: *Clinical Handbook* [18, 28]. Participants discussed how if health workers were not familiar with resources to inform appropriate care, they risk subjecting women to various negative experiences including stigma and bias during care (discussed later) and inappropriate interventions such as failing to address a Type 3 infibulation scar during labor and birth:

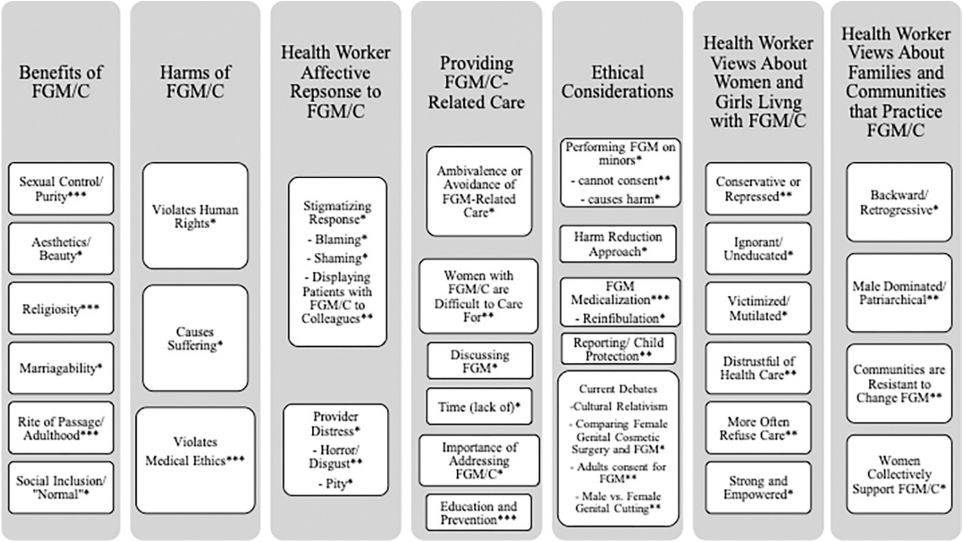


**Fig 2. Attitudes: Categories and codes.** *All Settings, **Low Prevalence, ***High Prevalence.

https://doi.org/10.1371/journal.pone.0284900.g002

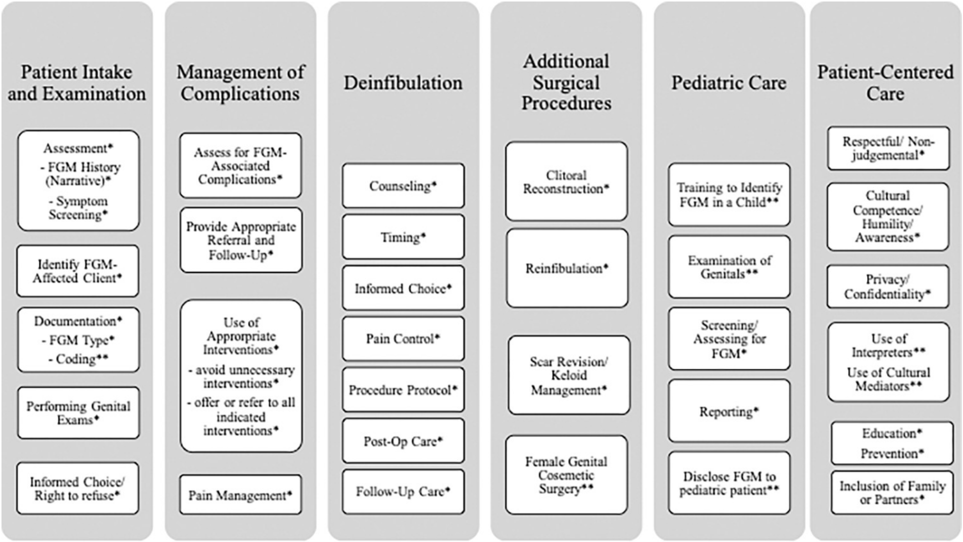


**Fig 3. Practices: Categories and codes.** *All Settings, ** Low Prevalence, ***High Prevalence.

https://doi.org/10.1371/journal.pone.0284900.g003

> "*Second stage of labor might be prolonged because of soft tissue distortion. Someone might not be aware of how to manage the scar. . . they should treat early in the second stage [with defibulation]. . . if you delay, second stage might be prolonged and it's likely [the fetus is] disposed for perinatal asphyxia. (High Prevalence)*

Participants stressed that health workers need opportunities to clarify their own attitudes toward the practice, women who have been affected by it, requests for FGM/C, defibulation, and reinfibulation (re-suturing a fully or partially opened type III scar, most commonly after childbirth) [29]. If this self-assessment is accomplished prior to interacting with affected patients, there is potential to more effectively and consistently empower patients as they navigate management options.

Given that there are legal implications for FGM/C, participants noted that health workers need to be aware of laws and reporting requirements in their own settings so they can act in accordance with local legal standards. At the same time, women living with FGM/C should not be treated as though they are the perpetrator; they should not be judged for something that was done when they were a child.

> *They should not be looked upon as criminals or those who had been subjected to harmful practice because they're not responsible for this. When they were subjected to this practice, they were unable to give their consent. They were not aware about the procedure. They were never consulted about the procedure. (High Prevalence)*

Participants, particularly from high prevalence settings, noted that many women who have undergone FGM/C do not identify as "victims" and do not want to be labelled as victims, but rather see themselves as strong and empowered women.

## Interactions between knowledge, attitudes and practices

In this section we describe the ways in which participants discussed how knowledge, attitudes and practices interact. As noted above, knowledge can inform clinical practice by enabling

health workers to assess for FGM/C, identify FGM/C type, assess for sequalae, provide treatment, and/or refer to specialist care. FGM/C-related knowledge can improve health workers' ability to counsel clients about the social or financial pressures to perform FGM/C and respond to requests to perform FGM/C or reinfibulation. If the learning does not occur, practices may not be in line with current evidence. While participants across settings uniformly agreed that FGM/C should not be performed, a participant described how a health worker's acceptance of FGM/C might change their practice:

> *A healthcare health worker coming from such communities. . . [probably supports] the traditional practice because of their socialization about FGM. . . if [they don't] feel like FGM is a big thing or, an infringement of human rights, or don't feel like it has a lot of health consequences, then they might easily overlook the kinds of problems that a woman has presented. (High Prevalence)*

Conversely, the clinical practice of a health worker who has negative attitudes toward FGM/C that manifest in their affect may be negatively impacted. For example, patients who experience stigma from the health worker because of the FGM/C may have a poor patient experience, and delay or avoid future care.

> *"If [a health worker] looks down upon my community and then I would say, "No, you are not the right health worker to give me care.". . . If I perceive you as not caring then why would I subject myself to [you] care? You wouldn't give me appropriate care, and I would perceive that your care is not well meant" (High Prevalence)*

All participants endorsed a patient-centered approach, insisting that health workers *earn* the patient's trust and not expect it based on their status as a health worker.

Participants discussed that attitudes toward FGM/C may motivate health workers to accept new knowledge about its harms, or resist learning about the harms of FGM/C. Participants across settings described how some health workers have negative attitudes towards communities where FGM/C is normative which may affect how care is delivered, i.e. by stigmatizing women and not treating them respectfully. In addition, subtle or overt expressions of bias may alienate patients. Attitudes or beliefs about women and communities that practice FGM/C may also lead health workers to make assumptions about patients. Participants across settings discussed how beliefs that women with FGM/C are less educated, backward or repressed (particularly related to sex and gender norms), or lack agency can result in patients feeling they are being looked down upon. Again, this can lead to avoidance of or discontinuing of care, either of which could result in poor outcomes. These assumptions may include preconceptions of how receptive a patient is to learning about FGM/C, possible treatments, FGM/C-related plans for any girl children and involvement from her partner or other family members. Some health workers may believe that a community's commitment to FGM/C is immutable, and therefore not even worth fighting against, further leading the health worker to avoid engagement with FGM/C prevention activities. Participants stressed the importance of listening to each individual patient and providing patient-centered care based on her priorities, not preconceptions about her or her community.

The typical KAP sequence is perhaps best illustrated by participants from both medium/ high and low prevalence countries who argued that understanding, but not endorsing, the perceived benefits of FGM/C may cultivate health workers' compassion and empathy, motivate learning about appropriate care, and enable them to more effectively care for and counsel patients without stigmatizing them and their families or communities. Arriving at an understanding of FGM/C's

perceived health benefits can occur in myriad ways–some participants described teaching this to colleagues and students. It can also occur organically. For example, a participant working in the low prevalence setting described how witnessing a nurse and patient from the same diasporic FGM/C practicing community led the participant to transform their attitudes toward FGM/C from one of pity for a perceived mutilation, toward an empathetic understanding of the meaning that the cutting may have for a woman. This participant underscored how empathy is not the same as support, and that even in the diasporic setting there may be health workers who support FGM/C as in a case where an operating room nurse from an FGM/C practicing community, upon seeing a Type 3 FGM/C during a cesarean birth, said:

> "'*This is so beautiful*!'. . . *that's when it really hit me that I need to honor women and really respect the value that they hold as it pertains to what's being done to their bodies that is not the Western lines of mutilation." (Low Prevalence)*

Several similar vignettes from low prevalence settings noted that many health workers may themselves be immigrants from places where FGM/C is normative and hold strongly positive views of FGM/C.

Lack of knowledge about FGM/C may lead to behaviors that are alienating as well. Participants in the medium/ high prevalence settings discussed how prevalence may vary by region within a country, and emphasized that health workers must show compassion even when they have a strong affective response against the practice:

> "*. . . Even if [they] don't understand [FGM/C] and are shocked and horrified*, *I think managing these kinds of emotions would be important because otherwise*, *it's going to create more reluctance [for the patient] to say more. (High Prevalence)*

Finally, participants discussed how a health workers' perception that caring for FGM/C is complex may discourage them from discussing it with the patient at all. This avoidance may lead to worsening of symptoms due to neglect. Participants described that women living with FGM/C that obstructs the introitus may not be offered routine pap smears or experience delays in obtaining procedures such as endometrial biopsy because of difficulty accessing internal structures. If avoidance takes the form of unnecessary interventions, iatrogenic complications can occur. A number of participants from low prevalence areas highlighted the overuse of cesarean birth as a mechanism some health workers use to avoid addressing complex discussions about defibulation and/or reinfibulation.

> *What about patients that request reinfibulation? [Some] health workers have ethical or moral reasons that they don't want to honor that. So, health workers end up offering a C-section because they say, "Oh, at least we can avoid dealing with that scar" (Low Prevalence)*

Participants also emphasized that women living with FGM/C have the right to refuse recommended care without fear of retribution and should never be forced or coerced to undergo any genital exams or procedures. Participants discussed how health workers must have sufficient knowledge to adequately counsel patients about recommended care, and have the communication skills to fully engage in shared decision-making rather than coercion.

## Discussion

Our analysis yielded six categories of knowledge, seven of attitudes, and six of practices important for the prevention and care of women and girls affected by FGM/C. Each category

includes additional codes as enumerated in Figs 1–3. We explored how knowledge, attitudes and practices interact in multi-directional ways to influence the type and quality of care provided to clients affected by FGM/C. Collectively, these data provide guidance for the development of items for knowledge, attitudes, and practices measurement tools.

With regards to the knowledge domain, our findings were consistent with earlier research confirming that foundational knowledge about FGM/C (including factors that drive the practice), being able to identify FGM/C, and knowledge of evidence-based care are important for the provision of quality care, and prevention of mismanagement [21, 23, 30–33]. High quality care can serve to build patient-provider trust, and trust in the health system [32]. With regards to FGM medicalization, KAP tools may need to capture the knowledge about laws, medical ethics and existing accountability mechanisms for those providers performing the practice themselves. In addition, the financial, cultural, or perceived religious pressures that may shape the attitudes and practices of health workers should be explored in the attitude component of a KAP tool [18, 34–36].

Our findings identified the inter-relation and the multi-directional influence among knowledge, attitudes and practice. We have found supportive and non-supportive attitudes towards FGM/C practice can lead to suboptimal care either through complacency, a negative judgmental attitude or an attitude that glorifies the practice, none of which represent a non-judgmental rights-based approach to care or respectful patient provider interaction. Research elsewhere has shown health workers' attitudes affect the quality of care and patient outcomes, increasing shame, stigma, and poor body self-image [32, 37, 38]. These negative affective responses may lead women and girls with FGM/C to delay or avoid seeking healthcare, and undermine trust of the health sector [39]. In the context of migration, stigmatization of women with FGM/C may impart additional burden on a patient who must emotionally prepare to face bias when seeking care [40, 41]. A recent systematic review of the experiences of care of women and girls who have undergone FGM/C found that they often experience disrespectful, unsafe and disempowering experiences of care–experiences that violate the human rights-based patient-centered care that all people deserve [42, 43]. It will be important to measure health workers' "self-awareness" or "stance on FGM/C" in KAP tools. Well-designed KAP measures will enable researchers to examine the inter-relation between attitudes and knowledge and practice, which will contribute to the design of better interventions and consequently better care and experiences for those accessing care.

Study participants emphasized that health workers must practice patient-centered care, communication skills, and build awareness of their own affective response (including verbal and non-verbal cues that communicate stigma and bias). Stigma and bias may include making assumptions about the beliefs and desires of a patient, or about those of their families or communities; stigma and bias can be communicated directly to patients or via health workers talk/ "sign-outs" or documentation [44]. Research demonstrates that health worker bias negatively affects quality of care, and can worsen patient health outcomes [32, 37, 38]. Participants also emphasized that women must freely consent to any procedures or exams, which is a basic standard of care. This emphasis is important to note given the context that women with FGM/C, specifically when they are pregnant or laboring, may be coerced or have their capacity to decline overruled by health workers [45].

FGM/C-affected women must be given the space to be heard, and providers must learn how to listen and build empathy. Resources are increasingly available to improve patient-centered communication and care in the context of FGM/C [46]. Longitudinal iterative experiential training and evaluation of patient care interactions have been demonstrated to provide useful feedback to health workers to assist in their self-reflection, growth and accountability for the care of marginalized and stigmatized groups [47]. These skills and practices would also

need to be assessed in a KAP tool; however, the measurement of these types of practices may be difficult to capture via self-report because they may be subject to social desirability bias.

Our study had strengths and limitations. Our study population included a broad sample of clinical and research experts representing a diversity of perspectives by discipline and regions, which contributes to the credibility of our study. The sampling, recruitment (including use of referrals) and profile of participants and interview guide may have weighted perspectives more toward higher income countries and on higher level of obstetric and gynecologic care of FGM/C survivors. There were no representatives from ministries of health who are responsible for setting standards and procedures within the public health systems in high prevalence countries. The resource constraints at health systems level in low resource settings affect the experiences and perspectives of providers in these settings yet they are underrepresented in this study. In addition, there is a lack of representation of perspectives from Asian countries. The sample includes participants from about a third of the 31 UNICEF-defined high FGM/C prevalence countries; however, a single participant from a country cannot represent the country as a whole. We presented a comprehensive framework of knowledge, attitudes, and practices but did not break down the identified knowledge and skills by specialty, competency or level of care (primary, secondary or tertiary level of care). Future research could further elaborate specific knowledge, attitudes and practices that are most relevant to particular regions, service levels, and health worker type.

Overall, our findings, irrespective of FGM/C prevalence, identified that adequate foundational knowledge, non-judgmental attitude and practice that is respectful and culturally sensitive and centered around the patient or client are pre-requisites for optimal care. The specifics or constructs of these domains will need to be designed according to the required competencies of health workers and the context in which they are trained and provide care. The categories and codes of knowledge, attitudes, and practices identified in this study can inform the development of measures to assess health worker knowledge, attitudes and practices for FGM/C prevention and care in specific settings. These measures would need to be psychometrically tested and validated. Measurement is a key component in evaluating pre- and in-service trainings of health workers and in designing and assessing programmatic interventions. Effective measurement of health worker KAP can drive greater accountability for the quality of care delivered, and eventually impact the health outcomes of women and girls living with FGM/C. This study is an important step in that process, with the ultimate goal of improving prevention and care services on FGM/C.

## Conclusion

Study participants described knowledge that health workers require in order to provide effective health care to women and girls affected by FGM/C, attitudes that may facilitate or impede the provision of FGM/C prevention and care, and the clinical practices that comprise safe, efficient, and accessible care. The categories and codes within knowledge, attitudes, and practices that were identified during our analysis will inform the development of an item bank which will inform the development of a comprehensive assessment tool of health worker knowledge, attitudes and practices.

## Acknowledgments

The authors would like to thank the participants, who generously shared their expertise and opinions with us.

## Ethics approval and consent to participate

The Johns Hopkins School of Medicine Institutional Review Board and WHO Ethical Review Committee granted ethical approval for this study. Oral informed consent was obtained from all participants. The interviewer explained the study aim and procedures to participants, confirmed their voluntary participation, and asked permission for audio recording before beginning all interviews. Identifying information was excluded from all transcribed texts to ensure anonymity of participants.

## Author Contributions

**Conceptualization:** Christina X. Marea, Nicole Warren, Nancy Glass, Wisal Ahmed, Christina C. Pallitto.

**Data curation:** Christina X. Marea.

**Formal analysis:** Christina X. Marea, Nicole Warren.

**Funding acquisition:** Christina X. Marea, Christina C. Pallitto.

**Investigation:** Christina X. Marea.

**Methodology:** Christina X. Marea, Nicole Warren, Nancy Glass, Christina C. Pallitto.

**Project administration:** Christina X. Marea.

**Resources:** Christina C. Pallitto.

**Supervision:** Nicole Warren, Nancy Glass, Christina C. Pallitto.

**Validation:** Christina C. Pallitto.

**Writing – original draft:** Christina X. Marea.

**Writing – review & editing:** Christina X. Marea, Nicole Warren, Nancy Glass, Wisal Ahmed, Christina C. Pallitto.

## References

1. Female Genital Mutilation (FGM) Statistics [Internet]. UNICEF DATA. [cited 2022 Apr 25]. Available from: https://data.unicef.org/topic/child-protection/female-genital-mutilation/
2. Relph S, Inamdar R, Singh H, Yoong W. Female genital mutilation/cutting: knowledge, attitude and training of health professionals in inner city London. Eur J Obstet Gynecol Reprod Biol. 2013 Jun; 168 (2):195–8. https://doi.org/10.1016/j.ejogrb.2013.01.004 PMID: 23434403
3. Kaplan A, Hechavarría S, Bernal M, Bonhoure I. Knowledge, attitudes and practices of female genital mutilation/cutting among health care professionals in The Gambia: a multiethnic study. BMC Public Health. 2013 Sep; 13(1):851. https://doi.org/10.1186/1471-2458-13-851 PMID: 24040762
4. Cappon S L'Ecluse C, Clays E, Tency I, Leye E. Female genital mutilation: Knowledge, attitude and practices of Flemish midwives. Midwifery. 2015; 31(3):e29–35. https://doi.org/10.1016/j.midw.2014.11.012 PMID: 25575861
5. Dawson A, Turkmani S, Fray S, Nanayakkara S, Varol N, Homer C. Evidence to inform education, training and supportive work environments for midwives involved in the care of women with female genital mutilation: A review of global experience. Midwifery. 2015; 31(1):229–38. https://doi.org/10.1016/j.midw.2014.08.012 PMID: 25246318
6. Purchase TCD, Lamoudi M, Colman S, Allen S, Latthe P, Jolly K. A survey on knowledge of female genital mutilation guidelines. Acta Obstet Gynecol Scand. 2013; 92(7):858–61. https://doi.org/10.1111/aogs.12144 PMID: 23581719
7. Sureshkumar P, Zurynski Y, Moloney S, Raman S, Varol N, Elliott EJ. Female genital mutilation: Survey of paediatricians' knowledge, attitudes and practice. Child Abuse Negl. 2016 May; 55(April):1–9. https://doi.org/10.1016/j.chiabu.2016.03.005 PMID: 27045807

8. Surico D, Amadori R, Gastaldo LB, Tinelli R, Surico N. Female genital cutting: a survey among health-care professionals in Italy. J Obstet Gynaecol J Inst Obstet Gynaecol. 2015 May; 35(4):393–6.
9. Van Baelen L, Ortensi L, Leye E. Estimates of first-generation women and girls with female genital mutilation in the European Union, Norway and Switzerland. Eur J Contracept Reprod Health Care. 2016 Nov 1; 21(6):474–82. https://doi.org/10.1080/13625187.2016.1234597 PMID: 27652839
10. Leye E, Mergaert L, Arnaut A, O'Brien Green S. Towards a better estimation of prevalence of female genital mutilation in the European Union: interpreting existing evidence in all EU Member States. Genus. 2014; 70(1):99–121.
11. Goldberg H, Stupp P, Okoroh E, Besera G, Goodman D, Danel I. Female Genital Mutilation / Cutting in the United States: Updated Estimates of Women and Girls at Risk, 2012. Public Health Rep. 2016; 131 (April):340–7. https://doi.org/10.1177/003335491613100218 PMID: 26957669
12. Barrett HR, Bedri N, Krishnapalan N. The Female Genital Mutilation (FGM)–migration matrix: The case of the Arab League Region. Health Care Women Int. 2021; 42(2):186–212. https://doi.org/10.1080/07399332.2020.1789642 PMID: 32903163
13. Shell-Duncan B, Naik R, Feldman-Jacobs C. A State-of-Art-Synthesis of Female Genital Mutilation/Cutting: What Do We Know Now? [Internet]. New York: Population Council; 2016. (Evidence to End FGM/C: Research to Help Women Thrive.). Available from: http://www.popcouncil.org/ EvidencetoEndFGM-C
14. Tordrup D, Bishop C, Green N, Petzold M, Vallejo FR, Vogel JP, et al. Economic burden of female genital mutilation in 27 high-prevalence countries. BMJ Glob Health. 2022 Feb; 7(2):e004512. https://doi.org/10.1136/bmjgh-2020-004512 PMID: 35105556
15. Abdulcadir J, Say L, Pallitto C. What do we know about assessing healthcare students and professionals ' knowledge, attitude and practice regarding female genital mutilation? A systematic review. Reprod Health. 2017;1–13.
16. Valente TW, Paredes P, Poppe PR. Matching the Message to the Process: The Relative Ordering of Knowledge, Attitudes, and Practices in Behavior Change Research. Hum Commun Res. 2006 Mar; 24 (3):366–85.
17. Albarracín D, McNatt PS. Maintenance and decay of past behavior influences: Anchoring attitudes on beliefs following inconsistent actions. Pers Soc Psychol Bull. 2005; 31(6):719–33. https://doi.org/10.1177/0146167204272180 PMID: 15833901
18. WHO. WHO | Care of girls and women living with female genital mutilation [Internet]. Who. 2018. 1–425 p. Available from: http://www.who.int/reproductivehealth/publications/health-care-girls-women-living-with-FGM/en/
19. Reig-Alcaraz M, Siles-González J, Solano-Ruiz C. A mixed-method synthesis of knowledge, experiences and attitudes of health professionals to Female Genital Mutilation. J Adv Nurs. 2016 Feb; 72 (2):245–60. https://doi.org/10.1111/jan.12823 PMID: 26434563
20. Zurynski Y, Sureshkumar P, Phu A, Elliott E. Female genital mutilation and cutting: a systematic literature review of health professionals' knowledge, attitudes and clinical practice. BMC Int Health Hum Rights. 2015 Dec; 15(1):32.
21. Dawson A, Homer CSE, Turkmani S, Black K, Varol N. A systematic review of doctors' experiences and needs to support the care of women with female genital mutilation. Int J Gynaecol Obstet Off Organ Int Fed Gynaecol Obstet. 2015 Oct; 131(1):35–40. https://doi.org/10.1016/j.ijgo.2015.04.033 PMID: 26118329
22. Dawson AJ, Turkmani S, Varol N, Nanayakkara S, Sullivan E, Homer CSE. Midwives' experiences of caring for women with female genital mutilation: Insights and ways forward for practice in Australia. Women Birth J Aust Coll Midwives. 2015 Sep; 28(3):207–14. https://doi.org/10.1016/j.wombi.2015.01.007 PMID: 25686876
23. Jordal M, Wahlberg A. Challenges in providing quality care for women with female genital cutting in Sweden–A literature review. Sex Reprod Healthc. 2018; 17(July):91–6. https://doi.org/10.1016/j.srhc.2018.07.002 PMID: 30193727
24. Marea CX, Warren N, Hopkins J, Glass N, Johnson-Agbakwu C, Perrin N. Assessing the Reliability and Validity of Attitudes and Condence Scales for the Care of Women and Girls Affected by Female Genital Mutilation/Cutting.: 1–28.
25. Mishel MH. Methodological studies: Instrument development. Adv Des Nurs Res. 1998; 2:238–84.
26. Group AMHR. Design, implementation, monitoring, and evaluation of mental health and psychosocial assistance programs for trauma survivors in low resource countries; a user's manual for researchers and program implementers. U S Johns Hopkins Univ Bloom Sch Public Health. 2013.
27. Hsieh HF, Shannon SE. Three Approaches to Qualitative Content Analysis. Qual Health Res. 2005 Nov; 15(9):1277–88. https://doi.org/10.1177/1049732305276687 PMID: 16204405

28. World Health Organization. WHO Guidelines on the management of health complications from female genital mutilation. 2016; 64.
29. UNAIDS Undp, UNFPA UNHCR, UNICEF UNIFEM, et al. Global strategy to stop health-care providers from performing female genital mutilation. World Health Organization; 2010.
30. Banks E, Meirik O, Farley T, Akande O, Bathija H, Ali M. Female genital mutilation and obstetric outcome: WHO collaborative prospective study in six African countries. Lancet. 2006; 367(9525):1835–41. https://doi.org/10.1016/S0140-6736(06)68805-3 PMID: 16753486
31. Varol N, Dawson A, Turkmani S, Hall JJ, Nanayakkara S, Jenkins G, et al. Obstetric outcomes for women with female genital mutilation at an Australian hospital, 2006–2012: A descriptive study. BMC Pregnancy Childbirth. 2016; 16(1):1–10. https://doi.org/10.1186/s12884-016-1123-5 PMID: 27793119
32. Hamid A, Grace KT, Warren N. A Meta-Synthesis of the Birth Experiences of African Immigrant Women Affected by Female Genital Cutting. J Midwifery Womens Health. 2018;1–11. https://doi.org/10.1111/jmwh.12708 PMID: 29569363
33. Berg RC, Odgaard-Jensen J, Fretheim A, Underland V, Vist G. An Updated Systematic Review and Meta-Analysis of the Obstetric Consequences of Female Genital Mutilation/Cutting. Obstet Gynecol Int. 2014; 2014:1–8. https://doi.org/10.1155/2014/542859 PMID: 25505915
34. Doucet MH, Pallitto C, Groleau D. Understanding the motivations of health-care providers in performing female genital mutilation: an integrative review of the literature. Reprod Health. 2017; 14(1):46. https://doi.org/10.1186/s12978-017-0306-5 PMID: 28335771
35. Koski A, Heymann J. Changes in support for the continuation of female genital mutilation/cutting and religious views on the practice in 19 countries. Glob Public Health. 2019; 14(5):696–708. https://doi.org/10.1080/17441692.2018.1542016 PMID: 30424713
36. El-Gibaly O, Aziz M, Hussein SA. Health care providers' and mothers' perceptions about the medicalization of female genital mutilation or cutting in Egypt: A cross-sectional qualitative study. BMC Int Health Hum Rights. 2019; 19(1):1–12.
37. Hagiwara N, Lafata JE, Mezuk B, Vrana SR, Fetters MD. Detecting implicit racial bias in provider communication behaviors to reduce disparities in healthcare: Challenges, solutions, and future directions for provider communication training. Patient Educ Couns. 2019; 102(9):1738–43. https://doi.org/10.1016/j.pec.2019.04.023 PMID: 31036330
38. Hall WJ, Chapman MV, Lee KM, Merino YM, Thomas TW, Payne BK, et al. Implicit racial/ethnic bias among health care professionals and its influence on health care outcomes: A systematic review. Am J Public Health. 2015; 105(12):e60–76. https://doi.org/10.2105/AJPH.2015.302903 PMID: 26469668
39. Evans C, Tweheyo R, McGarry J, Eldridge J, Albert J, Nkoyo V, et al. Improving care for women and girls who have undergone female genital mutilation/cutting: qualitative systematic reviews. Health Serv Deliv Res. 2019; 7(31):1–216.
40. Jacobson D, Grace D, Boddy J, Einstein G. Emotional Health Work of Women With Female Genital Cutting Prior to Reproductive Health Care Encounters. Qual Health Res. 2022 Jan; 32(1):108–20. https://doi.org/10.1177/10497323211049225 PMID: 34865565
41. Jacobson D, Glazer E, Mason R, Duplessis D, Blom K, Mont JD, et al. The lived experience of female genital cutting (FGC) in Somali-Canadian women's daily lives. PLoS ONE. 2018; 13(11):1–24. https://doi.org/10.1371/journal.pone.0206886 PMID: 30399181
42. Evans C, Tweheyo R, McGarry J, Eldridge J, Albert J, Nkoyo V, et al. Seeking culturally safe care: A qualitative systematic review of the healthcare experiences of women and girls who have undergone female genital mutilation/cutting. BMJ Open. 2019; 9(5).
43. Zampas C, Amin A, O'Hanlon L, Bjerregaard A, Mehrtash H, Khosla R, et al. Operationalizing a Human Rights-Based Approach to Address Mistreatment against Women during Childbirth. Health Hum Rights. 2020 Jun; 22(1):251–64. PMID: 32669805
44. Sun M, Oliwa T, Peek ME, Tung EL. Negative Patient Descriptors: Documenting Racial Bias In The Electronic Health Record: Study examines racial bias in the patient descriptors used in the electronic health record. Health Aff (Millwood). 2022 Feb 1; 41(2):203–11.
45. Kingma E, Porter L. Parental obligation and compelled caesarean section: careful analogies and reliable reasoning about individual cases. J Med Ethics. 2021 Apr; 47(4):280–6.
46. World Health Organization. Person-centred communication for female genital mutilation prevention: a facilitator's guide for training health-care providers [Internet]. Geneva: World Health Organization; 2022 [cited 2022 Sep 28]. Available from: https://apps.who.int/iris/handle/10665/351441
47. Keuroghlian AS, Mujugira A, Mayer KH. Healthcare worker training to improve quality of care for sexual and gender minority people in sub-Saharan Africa: learning from efforts in Uganda. J Int AIDS Soc [Internet]. 2021 Jul [cited 2022 Aug 31];24(S3). Available from: https://onlinelibrary.wiley.com/doi/10.1002/jia2.25728 PMID: 34189872
